# Microwave Three-Wave Mixing Spectroscopy of Chiral Molecules in Weakly Bound Complexes

**DOI:** 10.1021/acs.jpclett.3c01900

**Published:** 2023-08-11

**Authors:** Wenhao Sun, Melanie Schnell

**Affiliations:** † Deutsches Elektronen-Synchrotron DESY, Notkestr. 85, 22607 Hamburg, Germany; ‡ Institute of Physical Chemistry, Christian-Albrechts-Universität zu Kiel, Max-Eyth-Strasse 1, 24118 Kiel, Germany

## Abstract

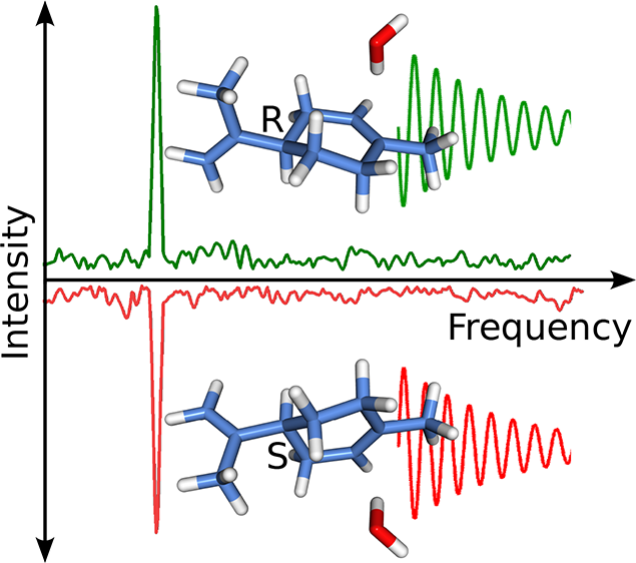

Since the first experimental implementation in 2013, microwave three-wave mixing has emerged as a robust spectroscopic approach for analyzing and controlling chiral molecules in the gas phase. This resonant, coherent, and nonlinear technique is based on the three-dimensional light–matter interaction in the electric dipole approximation, allowing for isomer- and conformer-selective chiral analysis with high resolution. Here we demonstrate the utility of microwave three-wave mixing for analyzing a molecular complex, limonene–H_2_O, which serves as a compelling example of addressing its potential to improve the chiral sensitivity for only weakly polar chiral molecules. The use of molecular complexes can also extend the applicability of microwave three-wave mixing to chiral systems that are not in the *C*_1_ point group.

Molecular chirality is an important concept in chemistry that refers to the property of molecules that cannot be superimposed on their mirror image. Chiral molecules are found throughout nature and play a crucial role in many areas of chemistry and biology.^1^ In living systems, a majority of biomolecules are chiral and encoded with a preferred “handedness”, such as right-handed (*R*) sugars and DNA and left-handed (*L*) amino acids in proteins.^2^ Therefore, biological environments often exhibit chirality and interact differently with the two enantiomers of the chiral guest molecules. One example is that *R*-carvone smells like spearmint to humans, whereas *S*-carvone smells like caraway seeds.^3^ This difference in interactions can also result in a stereoselective bias to chiral drug molecules, which comprise >50% of the drugs on the market today,^4^ as chiral receptor sites in biological systems like human bodies recognize enantiomers as different molecules and bind with only the one that has the proper absolute structural configuration.^5^ As such, new chiral drugs are designed to be enantioenriched, and asymmetric synthesis and enantiomeric purification have become significant topics in the modern chemistry and pharmaceutical industries. Despite the small difference caused by the parity-violating weak interactions,^6,7^ the physical properties of enantiomers are nearly identical, making chiral analysis a compelling but also challenging research topic.

Various specialized techniques have been developed to recognize, separate, and quantitatively analyze chiral molecules. In the field of chemistry, enantioselective chromatography-based techniques, including high-performance liquid chromatography (HPLC), gas chromatography (GC), and supercritical fluid chromatography (SFC), and capillary electrophoresis (CE) are those most often employed to achieve enantiomeric separations, and thereby chiral analysis.^8−10^ The employed chiral stationary phases and electrokinetic chromatography modes, which are designed on the basis of the enantiomer-specific interactions with auxiliary chiral substances, often require specific optimization for different chiral analytes. Additionally, spectroscopic methods, such as circular dichroism (CD) and vibrational circular dichroism (VCD),^11,12^ are widely applied in analyzing chiral samples in the solid and liquid/solution phases with the use of circularly polarized radiation. Chiral sum frequency generation (SFG) spectroscopy was developed to investigate chiral molecules at interfaces.^13,14^ Photoelectron circular dichroism (PECD) addresses molecular chirality in the gas phase.^15−17^ The gas-phase environment eliminates the solvent effects, enabling the high-resolution characterization of chiral molecules in an isomer- and conformer-selective manner.

Rotational spectroscopy, which has been used for decades to characterize structures of gas-phase molecules in great detail,^18^ can also be applied to study molecular chirality.^19−21^ Every molecular geometry has a unique rotational fingerprint. The transition frequencies depend on the three-dimensional mass distribution of the molecule in the inertial principal axis system, which is described by three rotational constants (*A*, *B*, and *C*), inversely proportional to the moments of inertia (*I*_*a*_, *I*_*b*_, and *I*_*c*_). The spectral strengths are proportional to the square of the magnitude of the electric dipole moment components (|μ_*a*_|, |μ_*b*_|, and |μ_*c*_|). Molecules, including their isomers, conformers, and isotopologues, can therefore be unambiguously differentiated and measured simultaneously. Especially since the invention of broadband chirped-pulse Fourier transform microwave (CP-FTMW) spectroscopy,^22^ rotational spectroscopy has become a robust and efficient technique for structural characterizations and shown great potential for other research and industrial applications, such as analyzing the composition of chemical mixtures and monitoring reaction processes.^23^ However, in terms of the enantiomers, they have the same moments of inertia and thus nearly identical rotational spectral features in addition to the small frequency differences arising from the parity-violating effects.^24^ They cannot be differentiated by conventional approaches for the measurement of microwave spectra.

To identify the enantiomers and perform quantitative chiral analysis, microwave chiral tagging^21^ and microwave three-wave mixing (M3WM) spectroscopy^19,25^ were developed. In microwave chiral tagging, the two enantiomers of the chiral analyte are converted into diastereomers by forming weakly bound molecular complexes with well-characterized chiral tag molecules. The resultant diastereomers are no longer mirror images and thus recognizable with rotational spectroscopy.^26^ In contrast, M3WM spectroscopy is an adaptation of conventional rotational spectroscopy, which has emerged as a nonlinear chirality-sensitive approach that can be used to probe the mirrored electric dipole allowed effects. In general, the chiral molecules are polarized with two orthogonal microwave fields, which drive two transitions in a cyclic three-level system. The three-level system is composed of all three types (a, b, and c) of rotational transitions, which are electric dipole allowed by μ_*a*_, μ_*b*_, and μ_*c*_, respectively. The two transitions are denoted as the “drive” and “twist” transitions. After the nonlinear excitations, a molecular response at the third (listen) transition frequency, which is not directly excited, is coherently induced in the third mutually orthogonal polarization direction and recorded as a function of time in the form of a free induction decay (FID). For enantiomers, this listen signal in the time domain has the same amplitude but its phase differs by π radians, as the triple product of the transition dipole moments [**μ**_***a***_·(**μ**_***b***_ × **μ**_***c***_)] of them has the same magnitude but the opposite sign. Thus, for an enantiomeric mixture, the amplitude of the listen signal is proportional to the enantiomeric excess (ee), and the signal can be expressed as
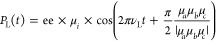
1where μ_*i*_ is the dipole moment component associated with the listen transition, ν_L_ is the frequency of the listen transition, and *t* is time. This approach can therefore be used for the quantitative determination of chiral compositions.^27^ For a racemic sample in which both enantiomers are present in equal amounts, the obtained signal will be zero.

A decade ago, the first M3WM experiment was successfully accomplished using 1,2-propanediol cooled with a cryogenic helium buffer gas [rotational temperature (*T*_rot_) ∼ 7 K], demonstrating that this method is capable of identifying enantiomers.^19,28^ Later on, our laboratory performed experiments using a microwave spectrometer with a supersonic jet, which cooled the gas-phase sample to a lower *T*_rot_ of ∼1–2 K.^29^ As rotational spectroscopy is sensitive to isomers and conformers, this approach can be used to analyze complex chemical samples containing multiple chiral species and molecules with multiple stereogenic centers.^20,30^ In addition to chiral analysis, it can further be applied to separate the two enantiomers in a specific rotational state with the inclusion of an additional excitation at the listen transition of the M3WM cycle, also known as enantiomer-specific state transfer.^31−34^ In this study, we present the results of an extended experiment in which we applied the M3WM method to investigate a weakly bound gas-phase complex, limonene–H_2_O (Figure 1). We also highlight the advantages of preparing chiral molecular complexes, particularly in cases in which the target molecule lacks sufficient electric dipole moment components.

**Figure 1:**
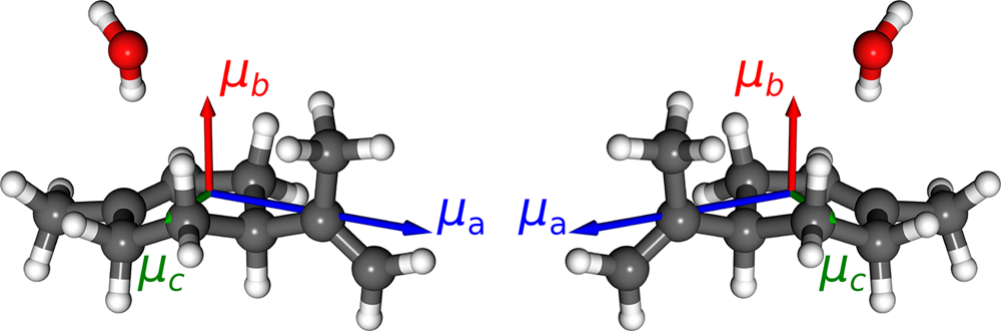
Isomer EQA-3 of monohydrated *S*-limonene (left) and *R*-limonene (right). The electric dipole moment components (μ_*a*_, μ_*b*_, and μ_*c*_) are indicated in the principal inertia axis system. The nomenclature, geometry, and the dipole moment components were taken from ref (35), which were computed at the B3LYP-D3(BJ)/6-311++G(d,p) level of theory.

The M3WM experiments were performed with the modified chirped pulse Fourier transform microwave (FTMW) spectrometer COMPACT (compact-passage-acquired coherence technique). The operation principle and the modifications have been reported in detail elsewhere.^36,37^ A brief description of the experiment follows. Both *R*- and *S*-limonene samples (chemical purity of 97%) are commercially available from Thermo Fisher Scientific and were used without further purification in this study. The limonene sample (melting point, −74 °C; boiling point, 176 °C) was placed in an internal reservoir and maintained at 50 °C. The reservoir is part of the pulsed solenoid valve (General valve Series 9) and located close to the valve orifice. Distilled water was held in a second reservoir installed upstream in the gas line outside the vacuum chamber of the spectrometer. Water and limonene vapor were seeded in helium carrier gas at a stagnation pressure of approximately 3 bar and supersonically expanded in the vacuum chamber via a pulsed valve operating at 6 Hz. The rotational temperature of the molecules was approximately 2 K in the gas jets.

The M3WM level scheme and pulse sequence are listed in Figure 2. The drive and twist microwave pulses were generated using a two-channel arbitrary waveform generator (AWG), amplified with two designated microwave amplifiers, a 40 W solid-state amplifier and a 300 W traveling-wave tube amplifier. After amplifications, the pulses were broadcast into the vacuum chamber via two horn antennas installed perpendicularly to each other. The molecular ensemble was polarized by the two back-to-back pulses, generating a enantiomer-sensitive molecular response at the listen frequency in the third mutually orthogonal direction. The time-domain free induction decay of this indirectly induced polarization was collected using a receiver horn on the detection side. For each gas pulse, the molecules were excited with six M3WM pulse sequences consecutively, leading to an effective repetition rate of 36 Hz. The collected molecular response was averaged by a digital oscilloscope and fast Fourier transformed to obtain the frequency-domain spectrum. The full width at half-maximum of the transition lines in the obtained spectrum is approximately 60 kHz. The high-resolution resonant characteristic of rotational spectroscopy allows us to perform the experiment in an isomer-selective way.

**Figure 2:**
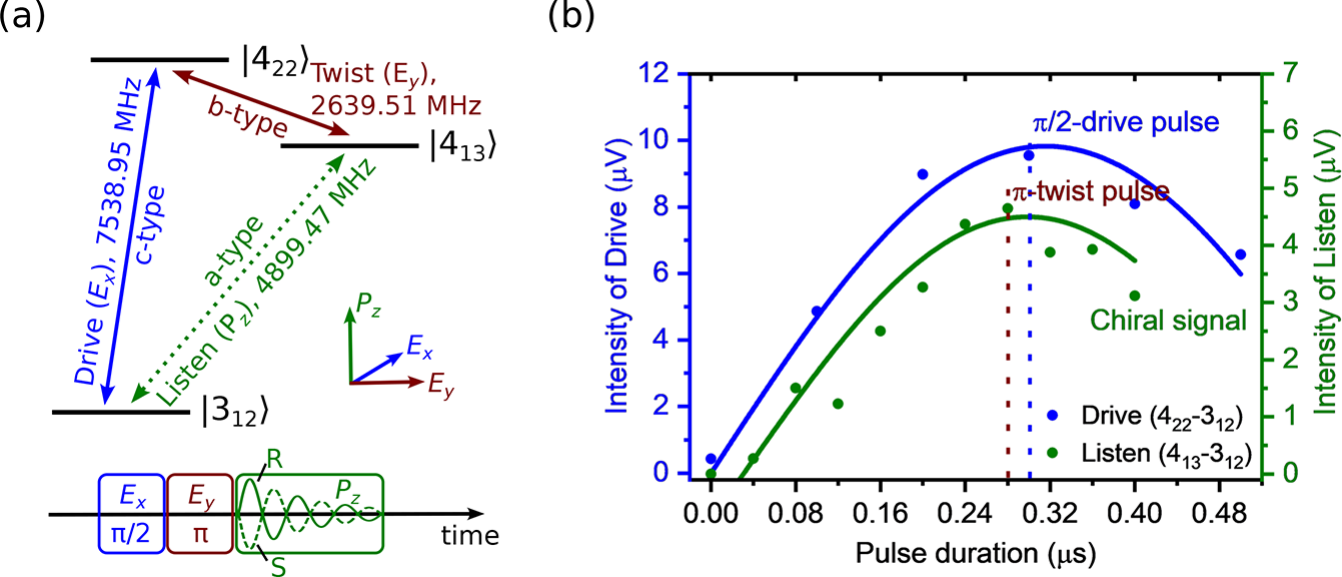
(a) M3WM level scheme and pulse sequence for the limonene–H_2_O complex: drive (c-type, 7538.95 MHz), twist (b-type, 2639.51 MHz), and listen (a-type, 4899.47 MHz). The rotational levels are denoted as  with *J* being the total angular momentum and *K*_*a*_ and *K*_*c*_ being the projections of this momentum onto the *a* and *c* principal axes, respectively, of the molecule. The spatial *M*_*J*_ degeneracies have been omitted for the sake of clarity.^41,42^ The associated microwave electric fields, *E*_*x*_ and *E*_*y*_, are orthogonal to each other in the laboratory frame, generating an indirectly induced polarization (*P*_*z*_) in the third mutually orthogonal direction. The transition frequencies were taken from the spectral assignment in ref (35). (b) Experimental nutation curves for optimizing the drive (blue) and twist (green) pulse conditions. The former excites the drive transition and directly monitors the spectral intensity at the drive frequency in the same polarization. The latter employs the M3WM scheme, which scans the pulse duration of the twist pulse, while the duration of the drive pulse is fixed at 0.3 μs, and monitors the intensity of the listen transition.

Previously, the broadband rotational spectrum of the limonene–water complexes has been studied in the frequency range of 2–8 GHz, and seven isomers of monohydrated limonene were explicitly assigned experimentally.^35^ The hydrogen-bonded complexes were produced through three-body collisions at the early stage of the supersonic jet expansion.^38,39^ Intrinsically, because water is a nonchiral interaction partner, the enantiomeric composition of the limonene–H_2_O isomers present in the supersonic jet should be all identical, which equals that in the limonene sample. Meanwhile, various other molecular species, such as pure water clusters, higher-order limonene–water complexes, and species from chemical impurities, can also be generated. Benefiting from the isomer selectivity of M3WM spectroscopy, we are capable of focusing on only the molecular system of interest, despite the presence of other species in the jets. For this proof-of-concept experiment, we focus on the most stable energetic isomer EQA-3 (see Figure 1). The rotational constants of EQA-3 have been well characterized: *A* = 1579.68818(50) MHz, *B* = 633.69037(29) MHz, and *C* = 553.80812(26) MHz.^35^ Accordingly, a suitable three-level system (|3_12_⟩ → |4_22_⟩ → |4_13_⟩) is selected for EQA-3 to be the M3WM scheme, as given in Figure 2a. Each rotational level is labeled as , where *J* is the total angular momentum quantum number and *K*_*a*_ and *K*_*c*_ are the projections of the angular momentum onto the *a* and *c* principal axes, respectively. The magnitudes of the associated electric dipole moment components are 1.6, 0.7, and 0.7 D for |μ_*a*_|, |μ_*b*_|, and |μ_*c*_|, respectively, predicted at the MP2/6-311++G(d,p) level of theory.^35^ As the intensity of the M3WM signal is proportional to the population difference between the two rotational states associated with the drive transition and the dipole moment component corresponding to the listen transition (see eq 1),^40^ the transition with the highest frequency, |3_12_⟩ – |4_22_⟩, is used as the drive transition and the a-type transition, |4_13_⟩ – |3_12_⟩, is used as the listen transition.

To achieve the optimal condition of the M3WM scheme, the durations of both drive and twist pulses were optimized by measuring the nutation curves, i.e., monitoring the respective transition intensity as a function of the pulse duration. For the drive pulse, the pulse duration was varied in steps of 0.1 μs. The excitation and detection are in the same polarization. As there is no direct detection installed in the same polarization with the twist pulse, the optimal duration of the twist pulse was obtained using the M3WM scheme by changing it in 0.04 μs steps, where the duration of the drive pulse was fixed at the optimal condition (0.3 μs). The chiral signal at the listen frequency was monitored, and the maximum was achieved at a twist pulse duration of 0.28 μs. The results are presented in Figure 2b. Note that these optimal pulse durations depend on the input power of the pulses. Afterward, the M3WM experiment was performed with both enantiomers using these optimized drive and twist pulse durations.

This process is also depicted by the Bloch sphere representation in Figure 3. Upon application of a resonant drive pulse, *E*_*x*_, at 7538.95 MHz, molecules initially populated in the |3_12_⟩ state are driven to a superposition state |+⟩^40,43^
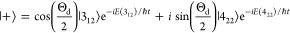
2where  is the Rabi flip angle of the drive pulse and relies on the strength and duration of the pulse, with μ_d_ being the transition dipole moment of the drive transition, |3_12_⟩ → |4_22_⟩. The maximum coherence between the |3_12_⟩ and |4_22_⟩ states is achieved at a Rabi flip angle of π/2, corresponding to a 50:50 split of the population difference between the two states. According to the nutation curve for the drive pulse (see Figure 2b), the effective π/2 condition is achieved at *t*_p_ = 0.3 μs, which averages over *M*_*J*_ substates of the rotational levels.^42^ Next, the induced coherence between the |3_12_⟩ and |4_22_⟩ states is transferred between the |3_12_⟩ and |4_13_⟩ states by applying a twist pulse, *E*_*y*_, in the polarization orthogonal to *E*_*x*_, which connects the |4_22_⟩ and |4_13_⟩ states and closes the cycle. This promotes molecules to the superposition state
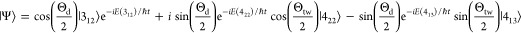
3where Θ_d_ and Θ_tw_ are the Rabi flip angles of the drive and twist pulses, respectively. The maximum transfer efficiency of the twist pulse is achieved at the π pulse condition, inverting the population in the |4_22_⟩ and |4_13_⟩ states.

**Figure 3:**
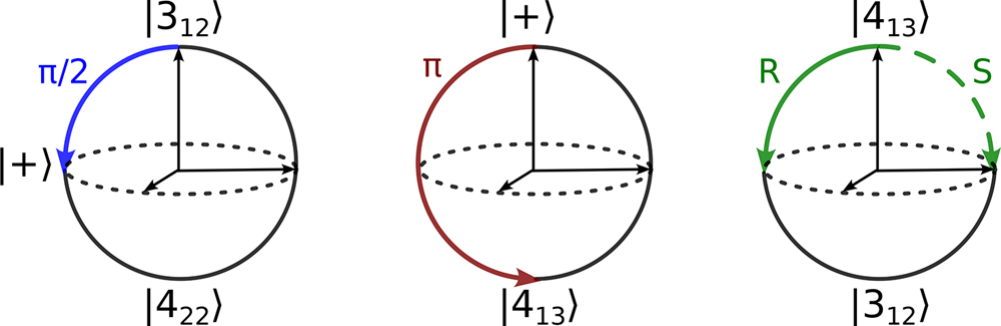
Bloch sphere representation of the M3WM scheme. The drive pulse creates coherence between the |4_22_⟩ and |3_12_⟩ rotational states, and the maximum coherence is achieved with a π/2 pulse. The coherence (|+⟩) is transferred between the |4_13_⟩ and |3_12_⟩ rotational states by applying a twist pulse between |4_22_⟩ and |4_13_⟩ at the π condition. Afterward, a chiral signal is indirectly induced at the listen frequency, the phase of which differs by π radians between the *R* (solid, green line) and *S* (dashed, green line) limonene–H_2_O complex.

After excitations with the drive and twist pulses, an indirectly induced coherence (*P*_*z*_) is obtained at the listen frequency (4899.47 MHz) in the polarization direction orthogonal to both *E*_*x*_ and *E*_*y*_ fields. When the drive and twist are π/2 and π pulses, respectively, the polarization in *P*_*z*_ can be simply described as in eq 1. As shown in Figure 2b, the chiral signal reaches a maximum intensity with a pulse duration of 0.28 μs, indicating the effective π condition of the twist pulse. Using the optimal conditions, the M3WM experiments were performed with both enantiomers in the monohydrated form, and the results are presented in Figure 4. A clear phase difference of π radians is observed for the two enantiomers, demonstrating that the M3WM technique can be used to identify the chirality of weakly bound molecular complexes. For a mixed sample in which both enantiomers are present, the enantiomeric compositions should be inherently identical in the liquid solution and vapor phase. As indicated in eq 1, the intensity of this listen signal obtained with such a sample is proportional to the enantiomeric excess of the molecular species of interest, allowing for quantitative analysis of chiral mixtures without enantiomeric separation.

**Figure 4:**
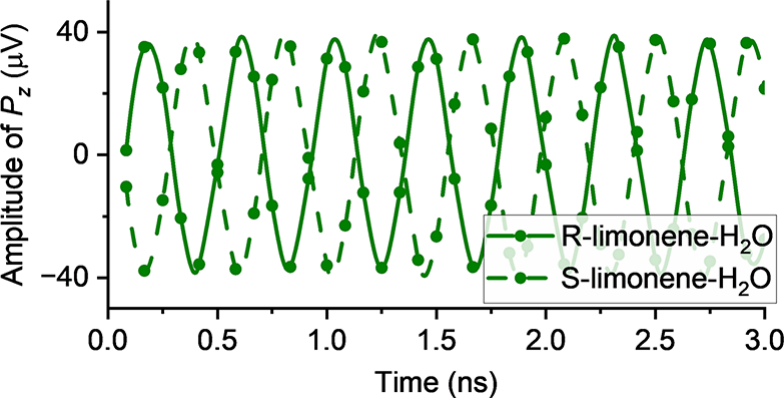
First 3 ns of the measured free induction decay at the listen frequency, 4489.47 MHz, for the *R* (solid) and *S* (dashed) limonene–H_2_O complexes.

In a previous microwave spectroscopic study, it was reported that the limonene monomer exhibits two stable conformers, EQ1 and EQ2, with the isopropenyl group in the equatorial (EQ) position.^44^ The two conformers have a difference in energy of approximately 1.2 kJ/mol, and their electric dipole moment components are all predicted to be <0.5 D. According to the quantum-chemical calculations at the MP2/6-311++G(2df,p) level of theory, for EQ1, |μ_*a*_| = 0.41 D, |μ_*b*_| = 0.37 D, and |μ_*c*_| = 0.35 D, while for EQ2, |μ_*a*_| = 0.35 D, |μ_*b*_| = 0.17 D, and |μ_*c*_| = 0.33 D. Because the M3WM signal relies on the electric dipole moment component allowing for the listen transition (see eq 1), this poses challenges for generating a sizable listen signal when analyzing limonene using M3WM spectroscopy. To address this issue, molecular complexes with polar partners can be employed to increase the overall electric dipole moment of the chiral system with the stereocenter preserved. In this study, by forming a complex with H_2_O, the monohydrated limonene, EQA-3, formed from monomer EQ1, has been predicted to exhibit electric dipole moment components of |μ_*a*_| = 1.6 D, |μ_*b*_| = 0.7 D, and |μ_*c*_| = 0.7 D, using the MP2/6-311++G(d,p) level of theory.^35^ The increased electric dipole moment of the one-water complex, particularly μ_*a*_, improves the sensitivity of the M3WM spectroscopy by a factor of 4 with a-type listen transitions, enabling a more efficient chiral analysis for limonene and other similar systems of interest. The combination of M3WM and molecular complexes offers a useful approach for analyzing chiral molecular systems, particularly for weakly polar chiral molecules like limonene. Compared to microwave chiral tagging, which also involves the analysis of molecular complexes, a notable difference of this method is that the tag molecules can be chiral and nonchiral. For example, this is convenient for direct analysis of the targeted chiral compounds obtained from chemical reactions, utilizing solvent molecules as binding tags and eliminating the need for sample purification.

Furthermore, there is another constraint of the M3WM method. It requires all three components of the electric dipole moment of the chiral molecule to be non-zero along the principal axes of inertia. Molecular symmetry dictates that molecules in the *C*_*n*_ and *D*_*n*_ point groups exhibit chiral geometries. In Figure 5, [4]helicene and twistane are provided as examples of chiral species in the *C*_2_ and *D*_2_ point groups, respectively.^45,46^ Among them, molecules in the *D*_*n*_ point group possess chirality but lack polarity, while those in the *C*_*n*_ point group are both chiral and polar. However, only those in the *C*_1_ point group can possess three non-zero dipole moment components. Consequently, the application of M3WM spectroscopy is exclusively limited to chiral molecules that possess *C*_1_ symmetry. To investigate chiral species belonging to the *C*_*n*_ (where *n* ≠ 1) and *D*_*n*_ point groups, the formation of complexes can be a useful and straightforward strategy for eliminating symmetry elements. By employing appropriate tag molecules, the resulting complexes can exhibit an overall *C*_1_ symmetry, thereby enabling chiral analysis using M3WM spectroscopy, while preserving the chiral configuration of the monomer.

**Figure 5:**
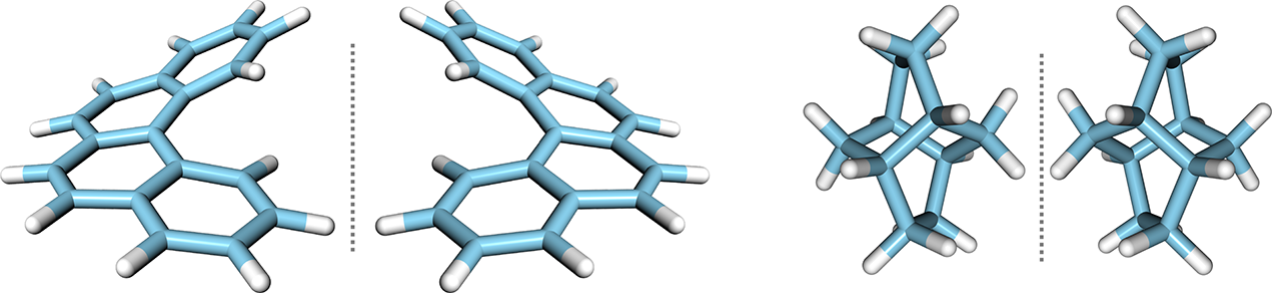
Molecular structures of the enantiomers of [4]helicene and twistane, which belong to the *C*_2_ and *D*_2_ point groups, respectively.

This approach also presents a convenient strategy for converting nonpolar nonchiral molecules into polar chiral complexes with the use of chiral tags. This holds important relevance, particularly for some systems of interest, such as the molecules containing heavy nuclei,^47^ which are commonly considered in the search for parity-violating effects.^48^ Upon formation of complexes with chiral tags, the synthesis of these molecules in a chiral form and the purification of their enantiomers are no longer needed, which will significantly simplify the experimental procedures. This approach will give researchers the freedom to explore a wider range of chiral molecular configurations and properties, without being limited to the constraints imposed by the traditional chiral synthesis. Moreover, it offers better control in experimental design. By incorporation of chiral tag molecules with diverse structural configurations and elemental compositions, researchers can meticulously investigate the interplay between the target molecule and various chiral environments. This enables a more efficient way to achieve a comprehensive understanding of the fundamental properties and behavior of molecules in a chiral context.

In conclusion, we have demonstrated the utility of the M3WM technique with a molecular complex, namely, limonene–H_2_O, in the gas phase. The isomer selectivity of M3WM spectroscopy allows us to investigate the isomer of interest, EQA-3, despite various other molecular species, such as the limonene monomer and other limonene–water complexes, being present in the supersonic jet. In the experiment, we successfully induced and probed a chiral signal at the listen frequency using the chosen M3WM cycle. The phase of the “listen” signal exhibited a shift of π radians between the two enantiomers. This result demonstrates that molecular chirality in weakly bound complexes can be probed by M3WM spectroscopy, thereby enabling their chiral analysis and further enantiomeric separation. The combination of M3WM with molecular complexes can facilitate the analysis of chiral molecules, particularly those with low electric dipole moments and high molecular symmetry (higher than *C*_1_). Using suitable binding partners, the formed weakly bound complexes as a whole can exhibit higher overall electric dipole moments and lower molecular symmetry compared to those of their monomeric counterparts while maintaining their chiral configurations at the stereogenic centers. This highlights the potential of M3WM spectroscopy to be extended to a broader range of chiral systems, offering a promising strategy for investigating their chirality. In addition, the produced chiral complexes can be enantio-separated in a specific rotational state using an enantiomer-selective state transfer scheme,^31−34^ which has been developed on the basis of the M3WM approach, preparing the enantio-enriched or enantio-purified samples for advanced precision experiments and further investigations.
